# Traumatic Septicæmia

**Published:** 1877-03

**Authors:** W. D. Boozer

**Affiliations:** Hogansville, Troup County, Ga.


### TRAUMATIC SEPTICÆMIA.

By W. D. BOOZER, Hogansville, Troup County, Ga.

On January 1st, 1877, at 3 P.M., was called to see a lady, æt. fifty-two. Four or five years ago had cancer removed from her nose, leaving scarcely any sign. On my arrival found her almost in spasms, suffering intensely of pain in the hand, with retching and vomiting, being in a perfect rigor. On the evening of previous day was injured by the bite of a calf, in attempting to unchoke it, contusing the hand or tissues on dorsum of metacarpal bones of the little and ring fingers, and lacerating a very small place beneath, at junction of the phalanx and metacarpal bone of little finger.

Hand gave but little trouble until this morning about ten o'clock, while working in the snow removing it from the shrubbery and flowers in the yard, when it began to pain her, and continued to get worse until my arrival. Seeing her condition, I proposed to use sulph. of morphia, hypodermically; but the patient objected, saying she once had it used, and came very near dying. I then gave a portion of morphia in connection with chloral hydrate. The stomach failing to retain these remedies after two trials of them, gave morphia sulph. without chloral. It being retained, the patient became a little more quiet, and in one hour I gave another dose, and made application to the contused part of pulv. opium, rubbed in lard, with a few drops of chloroform, the best I could select from my pocket-case. Patient became more quiet, with exception of twitching of muscles of the entire muscular system. I remained until nine o'clock; the patient being more quiet, I left a few powders of morphia to give along as needed. The hand was not swollen any, with exception of being a little puffed where contused; and thinking she would get along well, did not leave another engagement.

She did very well until late next evening, when the hand began to give pain and to swell, general rigors coming on. Twitching of muscles had not ceased; they becoming worse, she applied at night a poke-root poultice to the hand. Seeing the husband next morning, the 3d, he said she was not doing well, and that I had better call to see her. I did so, and found her still suffering with rigors and twitching of the muscles and dorsum of hand, blistered from poke-root poultice, and considerably swollen—tongue coated pretty heavy brown—quick pulse. I gave pretty free dose of mild chloride of mercury, with a little podophyllin; applied to the hand pulv. opium, with extract of belladonna rubbed in lard, and afterwards flax-seed and hop poultice.

I called next day at 10 A.M., and found patient a little more quiet, but still had twitching of the muscles; the hand and arm considerably swollen up to elbow, and discharge pretty free from the denuded surface of the hand. The medicine had acted well, and I continued administration of morphia and application of soothing poultices as long as swelling was intense; applied a weak solution of carbolic acid a few times, and administered mur. tr. iron and sulph. quinine three times per day.

On the 12th I made a pretty free incision into contused part of hand, letting out a quantity of pus, the patient complaining considerably of pain in the wrist joint. The arm was then swollen intensely to elbow, and red streak above elbow in region of brachial artery. The swelling gradually decreased in the forearm on 13th, but increased above elbow, with swelling of glands in axilla and very sore, considerably swollen up to the elbow. On 15th swelling ceased to extend any further, still abating in the forearm, but remained swollen above elbow until 18th, when the arm was very nearly the natural size, and tongue cleaning a little. The hand was discharging pus freely and healing slowly, but deep tissue sloughing at point where contused, forming cavity. A tent was kept in orifice for exit of pus. Patient complained every evening of rigors, etc.

On 21st had slight rigor, and on my arrival patient relating her condition, I examined to find some point of collection of pus or extension of disease, but could find only a tender point and a little tumifaction in the tissue between the metacarpal bones of the thumb and index finger. The tongue then of deep brown color. Two days later I made an opening at this point, and let out a small amount of pus, but next morning found on the shoulder some flush and a little œdema. I marked around with stick caustic, after which it did not spread but little, seemed to be about in patches, and gradually disappeared. The cautery I thought did no good, but was very severe.

About this time the patient received a very severe cut, about three inches, by breaking a *pou de chambre* It healed, however, without much trouble, but was very sore.

On the 30th discharged the patient, able to sit up several hours during the day; hand very nearly healed; gave directions to keep up the iron and quinine. The patient's appetite up to this time had increased but very little.

Was called February 10th to see another member of the family, when I examined her hand and found it not quite healed, owing to proud flesh. I made application of burnt alumn, and the same on the 11th.

On 27th, passing, saw patient, and found the hand healed but swollen, and fingers tolerably stiff, but can move them about a little; ring finger less than any, but will, I think, have pretty good use of the hand.

